# Multimodal Diagnostic Methods of Sepsis in Critically Ill Patients

**DOI:** 10.3390/diagnostics12081950

**Published:** 2022-08-12

**Authors:** Alexandru Florin Rogobete, Dorel Sandesc

**Affiliations:** 1 Faculty of Medicine, Victor Babes University of Medicine and Pharmacy, 300041 Timisoara, Romania; 2 Clinic of Anaeshtesia and Intensive Care, Emergency County Hospital Pius Brinzeu, 325100 Timisoara, Romania

The Special Issue “Multimodal Diagnostic Methods in Sepsis in the Critically Ill Patients” published in *Diagnostics*, Section “Pathology and Molecular Diagnostics”, reports a series of scientific works from varying international authors regarding different methods of diagnosis and identification of clinical signs in critical patients with sepsis. The Special Issue includes articles that describe in detail the expression of certain clinical scores and of some biomarkers, along with a series of correlations between them specific to patient with sepsis.

Tsai et al., in their original article “The Combination of SOFA Score and Urinary NGAL May Be an Effective Predictor for Ventilator Dependence among Critically Ill Surgical Patients: A Pilot Study”, analyzed the expression of gelatinase-associated lipocalin (NGAL), kidney injury molecule-1 (KIM-1), cystatin C, growth differentiation factor 15 (GDF-15), and calprotectin in mechanically ventilated critically ill patients and reported a series of correlations in relation to the clinical evolution of these patients. The study included 33 patients who were divided into two study groups (ventilator dependence and non-ventilator dependence). Following analysis, the authors reported a statistically strong correlation between NGAL expression and ventilator dependence. Moreover, the authors identified significant statistical correlations between the expression of the studied biomarkers and the SOFA and APACHE II scores [1].

Lee et al., in the article “The Association between Dynamic Changes in Serum Presepsin Levels and Mortality in Immunocompromised Patients with Sepsis: A Prospective Cohort Study”, analyzed presepsin changes in critically ill immunocompromised patients with sepsis. In the prospective study, 119 adult patients were included, whose presepsis level was monitored on day 1 after admission to the intensive care unit and on day 3 after admission. Following the study, the group of authors reported a higher concentration of presepsin on day 3 in patients who died in the hospital compared with those who survived. The conclusion of the study highlights the fact that changes in presepsin expression between day 1 and day 3 of admission to the ICU show direct and statistically strong correlations with the mortality rate [2].

Another article published in the Special Issue “Failure of Lactate Clearance Predicts the Outcome of Critically Ill Septic Patients” by Bruno et al. highlights a lack of correlations between lactate expression and the clinical prognosis of patients. The study included over 3000 critical patients and the analysis methods were formed by three models that took into account mechanical ventilation, certain clinical characteristics of the patients, and the treatment applied to the patients [3].

Valeanu et al., in a review entitled “Hemodynamic Monitoring in Sepsis—A Conceptual Framework of Macro- and Microcirculatory Alterations”, address and present in detail the impact of sepsis on the effects of microcirculation and on hemodynamic changes in critically ill patients. The article presents important physiopathological aspects regarding macrocirculation with essential elements from a clinical point of view regarding volemic resuscitation methods, complex monitoring methods, and hemodynamic principles. Additionally, the implications and importance of microcirculation monitoring in critically ill patient with sepsis are presented in detail [4].

Critical patients with sepsis always represent a challenge for intensive care doctors, representing a multifactorial complex of biochemical, molecular and clinical effects that make the clinical evolution unpredictable most of the time. In current clinical practice, a series of diagnostic and monitoring methods are used, but numerous literature studies present a series of modern biomarkers for quick and early detection of specific phenomena. Important to emphasize is the positive impact on the clinical prognosis that multimodal monitoring can bring, both of the biochemical pathways and of the hemodynamics, ventilator dependence and nutritional clinical effects [5,6,7,8].

## Data Availability

Not applicable.
